# A Homœopathic Materia Medica on a New and Original Plan

**Published:** 1895-06

**Authors:** 


### A Homœopathic Materia Medica on a New and Original Plan. By M. W. Van Denburg, M. D. A Sample Fascicle, containing the Arsenic group. Published by the author: Fort Edward, New York, U. S. A., 1895. Price, $1.50.

Dr. Van Denburg, a well-known homœopathic physician, and prominent member of the American Institute of Homœopathy, has for years been engaged in the building up of a materia medica on a plan which was conceived by him long ago, and which renders it the most perfect thing of the kind yet brought before the profession.

With a view of meeting the interminable controversies that have arisen about the authenticity of the symptoms of the materia medica, the author has sought to give the authority of every symptom, the name of the prover, and the publication where found.

All this makes a very elaborate symptomatology of highest value to the philosophic student, but unsuitable for ready reference when studying a case. To meet this defect another section is given where the symptoms are detailed in the ordinary way of other works on materia medica.

Then still another section is arranged where only the great characteristics or keynotes are given, and so the book is adapted to meet the wants of all classes of students of materia medica. The book rejects nothing, advocates nothing; but with scrupulous fidelity gives every symptom known, traces it to its sources, and gives the clearest ideas how it came into the materia medica. The whole arrangement is the most elaborate yet attempted, and withal most satisfactory.

The author expects to publish it himself if he can secure one thousand subscribers. This he ought easily to accomplish among the great body of workers in Homœopathy. The sample pages furnished, and now under review in this article, do not, in our opinion, do the author justice. The conception of the book is even finer than he has shown in the printed sample. Dr. Van Denburg paid a visit to this office and exhibited his manuscript. It made a most favorable impression upon the editor of this journal, and he has not forgotten it; but, on the contrary, has sought, in frequent conversations with various members of the profession, to inspire in them a friendly interest in so valuable an enterprise for the good of the cause.

It may be said that we have all that is needed in the *Encyclopædia of Materia Medica* and in the *Guiding Symptoms*. In answer to this, we can say that it covers the fields of both these publications, and goes over still more ground than they comprise. It does not supplant them, but rather is a sequel to them and an addition.

We cordially commend the work to the profession, and hope every one of our own subscribers will send in his name to Dr. Van Denburg, and thus insure the completion of the work.

